# The novel anti-cancer fluoropyrimidine NUC-3373 is a potent inhibitor of thymidylate synthase and an effective DNA-damaging agent

**DOI:** 10.1007/s00280-023-04528-5

**Published:** 2023-03-31

**Authors:** Jennifer Bré, Alison L. Dickson, Oliver J. Read, Ying Zhang, Fiona G. McKissock, Peter Mullen, Peijun Tang, Greice M. Zickuhr, Clarissa M. Czekster, David J. Harrison

**Affiliations:** 1 School of Medicine, University of St Andrews, North Haugh, St Andrews, KY16 9TF UK; 2 School of Biology, University of St Andrews, North Haugh, St Andrews, KY16 9ST UK; 3 NuCana Plc, 3 Lochside Way, Edinburgh, EH12 9DT UK

**Keywords:** Fluoropyrimidine, Colorectal cancer, Thymidylate synthase, DNA damage, NUC-3373

## Abstract

**Introduction:**

Fluoropyrimidines, principally 5-fluorouracil (5-FU), remain a key component of chemotherapy regimens for multiple cancer types, in particular colorectal and other gastrointestinal malignancies. To overcome key limitations and pharmacologic challenges that hinder the clinical utility of 5-FU, NUC-3373, a phosphoramidate transformation of 5-fluorodeoxyuridine, was designed to improve the efficacy and safety profile as well as the administration challenges associated with 5-FU.

**Methods:**

Human colorectal cancer cell lines HCT116 and SW480 were treated with sub-IC_50_ doses of NUC-3373 or 5-FU. Intracellular activation was measured by LC–MS. Western blot was performed to determine binding of the active anti-cancer metabolite FdUMP to thymidylate synthase (TS) and DNA damage.

**Results:**

We demonstrated that NUC-3373 generates more FdUMP than 5-FU, resulting in a more potent inhibition of TS, DNA misincorporation and subsequent cell cycle arrest and DNA damage in vitro. Unlike 5-FU, the thymineless death induced by NUC-3373 was rescued by the concurrent addition of exogenous thymidine. 5-FU cytotoxicity, however, was only reversed by supplementation with uridine, a treatment used to reduce 5-FU-induced toxicities in the clinic. This is in line with our findings that 5-FU generates FUTP which is incorporated into RNA, a mechanism known to underlie the myelosuppression and gastrointestinal inflammation associated with 5-FU.

**Conclusion:**

Taken together, these results highlight key differences between NUC-3373 and 5-FU that are driven by the anti-cancer metabolites generated. NUC-3373 is a potent inhibitor of TS that also causes DNA-directed damage. These data support the preliminary clinical evidence that suggest NUC-3373 has a favorable safety profile in patients.

**Supplementary Information:**

The online version contains supplementary material available at 10.1007/s00280-023-04528-5.

## Introduction

For 60 years, 5-fluorouracil (5-FU) has remained one of the most widely prescribed chemotherapies, used to treat common cancers including colorectal, gastric, breast, pancreatic, and head and neck. 5-FU exerts its anti-cancer activity through several key metabolites; fluorouridine triphosphate (FUTP), fluorodeoxyuridine monophosphate (FdUMP) and fluorodeoxyuridine triphosphate (FdUTP). FUTP is misincorporated into RNA instead of uridine [1] causing alterations in RNA processing and function. FdUMP inhibits deoxythymidine monophosphate (dTMP) synthesis through the formation of a covalent ternary complex with thymidylate synthase (TS) and 5,10-methylenetetrahydrofolate [1, 2], preventing the conversion of deoxyuridine monophosphate (dUMP) to dTMP. This imbalance in the ratio of dUMP to dTMP causes higher uracil incorporation in DNA, leading to cell cycle arrest and cell death. FdUTP is incorporated into DNA instead of deoxythymidine triphosphate, resulting in DNA damage.

The clinical effectiveness of 5-FU is limited by shortcomings associated with breakdown and activation. Most (> 85%) administered 5-FU is degraded by the enzyme dihydropyrimidine dehydrogenase (DPD) in the liver, generating alpha-fluoro-beta-alanine (FBAL) [2], a catabolite associated with off-target toxicities such as hand-foot syndrome and cardiotoxicity [3–5]. Therefore, most of the drug is catabolized before it can enter cancer cells and exert any therapeutic effect. The 5-FU taken up by cancer cells is dependent on expression of thymidine phosphorylase and thymidine kinase for conversion to fluorodeoxyuridine (FUDR) and phosphorylation to FdUMP. Thus, alteration in the levels of these enzymes limits the anti-cancer activity of 5-FU [6, 7]. Furthermore, misincorporation of FUTP in RNA causes myelosuppression, diarrhea and mucositis. To shift the metabolite profile from FUTP to FdUMP and limit toxicities, 5-FU is typically administered over long infusion times, up to 46 h [9]. Although 5-FU-based regimens are the standard of care for metastatic colorectal cancer (CRC), treatment failure is observed in over 90% of patients due to limited efficacy combined with systemic toxicity [8]. This highlights the urgent need for new therapies that improve the benefit-risk ratio for patients.

NUC-3373 is a phosphoramidate transformation of FUDR, comprised of FUDR and a phosphoramidate moiety (consisting of a phosphate and a specific combination of aryl, ester and amino acid groups) [7, 9]. The phosphoramidate moiety protects the molecule from DPD-mediated degradation, conferring the advantage of reduced exposure to toxic catabolites and associated toxicities, as well as significantly prolonging the plasma half-life (6–10 h for NUC-3373 versus 8–14 min for 5-FU) [10–12]. Owing to improved pharmacokinetics and direct delivery of the active metabolite FdUMP, NUC-3373 can be administered over a much shorter infusion compared to 5-FU (2 h versus 46 h) [13]. Thus, NUC-3373 has a more predictable metabolic pathway and is anticipated to improve the efficacy and safety profile, as well as reducing the administration burdens, that limit the clinical utility of 5-FU.

A Phase Ib/II study (NuTide:302) of NUC-3373 in combination with standard agents used for the treatment of advanced CRC is underway (NCT03428958). Although this study is designed to determine the recommended Phase II dose and assess safety, promising signals of anti-cancer activity have been observed in heavily pre-treated patients who are refractory to, or have relapsed on, prior fluoropyrimidine therapy [10]. Data from this study also support that NUC-3373 is associated with a lower incidence and severity of typical fluoropyrimidine-related toxicities (neutropenia, mucositis, diarrhea and hand-foot syndrome), compared to historical data for 5-FU. Therefore, it is important to establish the underlying cellular and molecular mechanisms responsible for these observations.

We hypothesized that differences in the levels of active metabolites generated following 5-FU and NUC-3373 administration could lead to a more precise mode of action. Here, we assess the differences between 5-FU and NUC-3373 in CRC in vitro, utilizing a model that mimics a short infusion rather than a prolonged continuous infusion, and discuss how these may contribute to an improved benefit-risk profile of NUC-3373.

## Materials and methods

### Cell culture and reagents

HCT116 and SW480 cell lines were purchased from ECACC and cultured in Dulbecco’s Modified Eagle Medium (DMEM—Gibco) with 10% (v/v) fetal bovine serum and 1% (v/v) Penicillin/Streptomycin. Cells were incubated at 37 °C with 5% CO_2_. They tested negative for Mycoplasma using the Minerva Biolabs ‘Venor GeM One Step’ PCR kit.

NUC-3373 was supplied as powder by NuCana plc, all other compounds were obtained from Sigma Aldrich. NUC-3373 and 5-FU were dissolved in DMSO to concentrations of 40 mM and 10 mM. Uridine and thymidine were dissolved in distilled water to concentrations of 10 mM and 4.15 mM, respectively. All stock solutions were aliquoted and stored at − 20 °C.

### Cell growth assays

Cells were seeded in 96-well plates at a density of 500 cells (HCT116) and 1500 cells (SW480) per well in a final volume of 200 µL/well in six experimental replicates and left to settle for 48 h prior to treatment. CRC cells were incubated with culture media containing increasing concentrations of NUC-3373 or 5-FU, with or without 1 mM uridine or 33 µM thymidine. After 24 h, drug-containing media was replaced with fresh media. The IC_50_ for each experiment was determined at 96 h post-treatment, using a Sulforhodamine B (SRB) assay[14]. Plates were scanned using BioTek 800 HT plate reader (540 nm absorbance filter) and data analysis was performed with Graphpad prism.

### Western blot

2 × 10^5^ HCT116 cells and 3 × 10^5^ SW480 cells were plated in 10 cm dishes and left for 48 h prior to treatment with different concentrations of NUC-3373 or 5-FU for 6 h. Cells were washed in ice-cold PBS and lysed in RIPA buffer supplemented with cOmplete mini protease inhibitor (Roche), aprotinin (Sigma Aldrich), and phosphatase inhibitor cocktails 2 & 3 (Sigma Aldrich). Protein concentration in each lysate was determined by Bicinchoninic Acid assay using Pierce BCA protein assay kit (Thermo Scientific, UK). Lysates (25 µg) were resolved by SDS Polyacrylamide Gel Electrophoresis, alongside Chameleon Duo protein ladder (LI-COR), then transferred to a PVDF membrane (Millipore) overnight at 4 °C. The membrane was blocked with 50% (v/v) Odyssey Blocking Buffer (LI-COR) in PBS for 1 h at RT. Antibodies for TS (Abcam #108995, 1:1000), ß-Actin (CST #3700 s, 1:10,000), γH2AX (CST #9718, 1:1000) and p-Chk1 (CST #2348, 1:1000) were diluted in 50% (v/v) Odyssey Blocking Buffer in PBS prior to incubation with the membrane. Next, membranes were incubated with Licor IRDye 800CW donkey anti-rabbit and IRDye 680RD donkey anti-mouse antibodies (1:10,000). After secondary antibody incubation and washes, membranes were dried in the dark and imaged using Odyssey® CLx Imaging System.

### Metabolite analysis by LC–MS and LC–MS/MS

#### Sample preparation for intracellular metabolites

2 × 10^5^ HCT116 cells or 3 × 10^5^ SW480 cells were plated in 10 cm dishes and left for 48 h prior to treatment with different concentrations of NUC-3373 or 5-FU for 6 h. Cells were washed, trypsinized, spun down and the supernatant discarded. The pellets were resuspended in 1 mL PBS and transferred to an Eppendorf tube for another centrifugation step. The PBS was discarded and pellets resuspended in 500 µL 80% ice-cold LC–MS grade methanol, vortexed and incubated at − 80 °C for 20 min. Samples were spun down at full speed for 5 min, 480 µL of supernatant transferred to a fresh tube and stored at − 80 °C until LC–MS analysis. Cell pellets were kept at − 20 °C for protein quantification.

Control matrix (cell supernatant) was prepared by extracting untreated HCT116 and SW480 cells as above. Internal standard solution containing ^13^C_10_^15^N_5_-dATP, ^15^N_5_-AMP and NUC-1031 was prepared in 20% acetonitrile (ACN) to a concentration of 20 µM, 200 µM and 2 nM respectively. Calibration standards were prepared for FdUMP, dUMP, FUTP and NUC-3373 in cell supernatant across the range 5.00–2000 nM, 50.0–20,000 nM, 1- 400 nM and 0.05–20 nM respectively. 10 µL of internal standard solution was added to each cell supernatant sample and calibration standard. Samples were vortexed and evaporated to dryness under a stream of nitrogen gas, then reconstituted in 75 µL of 20% ACN and analyzed by LC–MS.

#### LC–MS analysis

LC–MS analysis carried out on Acquity H-Class UPLC system coupled to Waters Xevo G2-XS Q-TOF. Analysis was carried out by electrospray ionization (ESI) source operated in either positive or negative ionization mode. Each sample was injected under three separate analytical conditions and analyzed in either full scan TOF mode (50–800 m*/z*) or by targeted analysis using multiple reaction monitoring (MRM). All solvents and buffers were of LC–MS grade. Chromatographic parameters can be found in supplementary information.

#### Mass spectrometer

The eluent from the LC system was infused directly into the ESI source. FUTP and FUDR were analyzed by an accurate mass full scan method due to specificity problems. FdUMP, dUMP, NUC-3373 and FUR were analyzed by targeted MRM. The conditions and *m/z* for each analyte is detailed in supplementary information (Table S2).

#### Calculations and normalization

Calibration lines were plotted linear 1/x^2^ and analyte concentration was calculated either on analyte area or peak area ratio with internal standard using MassLynx (version 4.2). The concentration of each analyte was converted to pmol. For each cell line, the protein content of a million cells was established: HCT116 130 µg/10^6^ cells; SW480 134 µg/10^6^ cells. Sample pellets (see sample preparation) were resuspended in 2% SDS and BCA was performed to determine protein concentration in each sample. This was used as a surrogate to calculate number of cells and results are reported as pmol/10^6^ cells. Areas under the curve were determined on Graphpad Prism.

### Flow cytometry

7.5 × 10^4^ HCT116 cells and 1.3 × 10^5^ SW480 cells were plated in 6 cm dishes and left for 48 h prior to treatment with different concentrations of NUC-3373 or 5-FU for 6 h. Cells were trypsinized and centrifuged at 1200 rpm for 5 min, washed with PBS and centrifuged again before being re-suspended in 1 mL of ice-cold 70% ethanol and stored at − 20 °C until staining for flow cytometric analysis. Samples were labeled with DAPI (1:3700) in PBS. Flow cytometry was performed using a CytoFlex (Beckman Coulter) and data analyzed using CytExpert (ver 2.4). G1 peak was identified based on the first peak of DAPI fluorescence, and G2 was measured as double level of fluorescence.

### Assay of dUTPase activity detected by EnzChek pyrophosphate assay kit

The activity of purified dUTPase was measured using EnzChek pyrophosphate assay kit. For dUTP and FdUTP substrate kinetic determination the assays contained: 10 nM dUTPase, 1 U purine nucleoside phosphorylase, 0.03 U inorganic pyrophosphatase, 200 μM MESG substrate, 0–50 μM of dUTP or 0–60 μM of FdUTP in reaction buffer (50 mM Tris–HCl, 1 mM MgCl2, pH 7.5). For dUMP inhibition study, dUTP concentration varied from 0–100 μM, dUTPase concentration was 20 nM and dUMP concentrations were 10 μM, 25 μM and 50 μM. For FdUMP inhibition, dUTP concentration varied from 0 to 100 μM, 50 nM of dUTPase was used when 10 μM of FdUMP was included, 100 nM of dUTPase was used when 25 μM FdUMP was included, and 200 nM of dUTPase was used when 50 μM FdUMP was included. The mixture without dUTPase was pre-incubated for 10 min to reduce background before enzyme was added to start reaction measurements. The reaction was performed at RT for 15 min while absorbance was monitored at 360 nm. Values were determined with reference to pyrophosphate standards provided by the kit. Only reactions under initial rate considered, data were fitted using a Michaelis Menten Eq. (1) and an equation for competitive inhibition (2).

1 $$k_{cat} = {\raise0.7ex\hbox{${V_{\max } }$} \!\mathord{\left/ {\vphantom {{V_{\max } } {E_{t} }}}\right.\kern-0pt} \!\lower0.7ex\hbox{${E_{t} }$}},\quad {\text{and}}\quad v_{i} = \frac{{k_{{{\text{cat}}}} \left[ S \right]}}{{K_{M} + \left[ S \right]}},$$

2 $${v}_{i}= \frac{{k}_{cat}[S]}{{K}_{M}(1+\left([I]/{K}_{i}\right))+[S]}$$

where *k*_cat_ is the turnover number, *V*_max_ is maximum velocity, E_t_ is total enzyme concentration, [S] is the concentration of substrate, *K*_M_ is the Michaelis constant, [*I*] is the concentration of inhibitor, *K*_i_ is the inhibition constant.

### Data availability

The data generated in this study are available upon request from the corresponding author.

## Results

### NUC-3373 causes a greater increase in TS complex formation in CRC cells compared to 5-FU

**Fig. 1:**
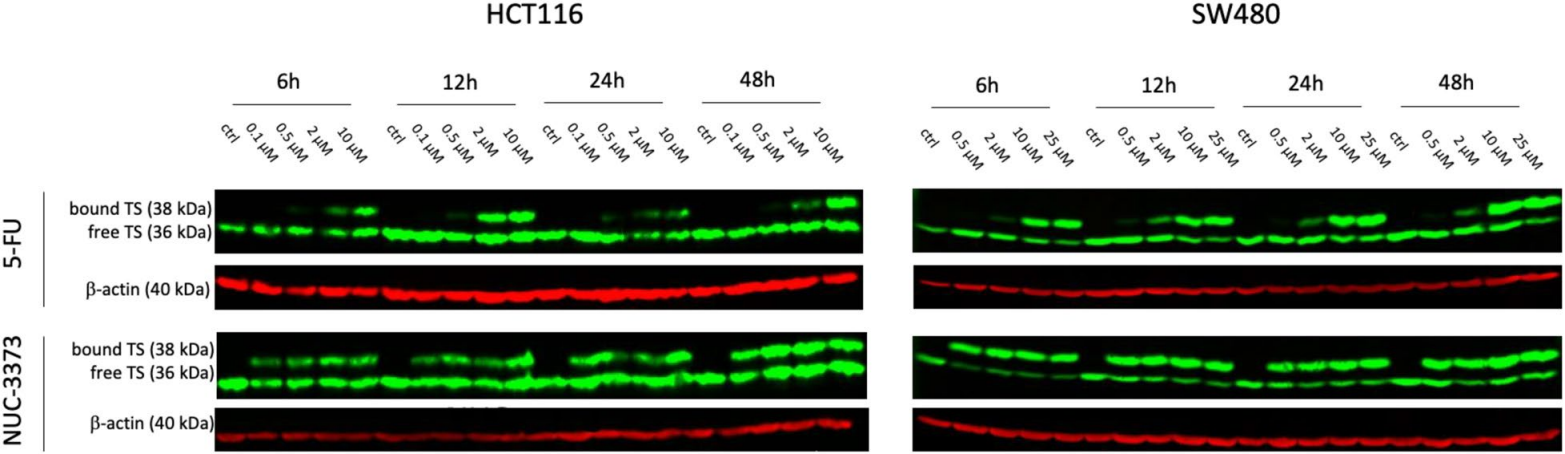
NUC-3373 and 5-FU cause formation of the TS ternary complex in CRC cells. Representative western blot images of TS expression at different timepoints in HCT116 (left) and SW480 (right) cells treated with a range of 5-FU (top) or NUC-3373 (bottom) doses (0.1–25 μM). The binding of FdUMP to TS, causing inhibition of the enzyme, is determined by the presence of an upper band on western blot (unbound TS 36 kDa; bound TS 38 kDa). β-actin was used as a loading control (*n* = 2)

TS ternary complex can be visualized on western blot as an indicator of inhibition through a shift of TS to a higher molecular weight at 38 kDa (free TS detected at 36 kDa). This method was established by Johnston et al. (1991) and confirmed by a number of research studies [15–17]. Two human colorectal cell lines HCT116 (microsatellite instable, MSI) and SW480 (microsatellite stable, MSS) were chosen based on their sensitivity to fluoropyrimidines and treated with equimolar sub-IC_50_ doses of NUC-3373 or 5-FU (0.1 μM to 25 μM) for 6 h (Table S1, Fig. S2). The dynamics of TS binding were assessed over time by the ratio of ternary TS complex to total TS (Fig. 1). In both cell lines, NUC-3373 led to a higher proportion of bound TS protein at low drug concentrations. Indeed, 10 μM of 5-FU was required to achieve the same level of TS binding as 0.1 μM of NUC-3373 in HCT116 cells and as 0.5 μM of NUC-3373 in SW480 cells. The binding of TS by NUC-3373 was almost maximal by 6 h and was sustained for at least 48 h in both cell lines.

### NUC-3373 generates higher levels of active anti-cancer metabolite FdUMP compared to 5-FU

**Fig. 2:**
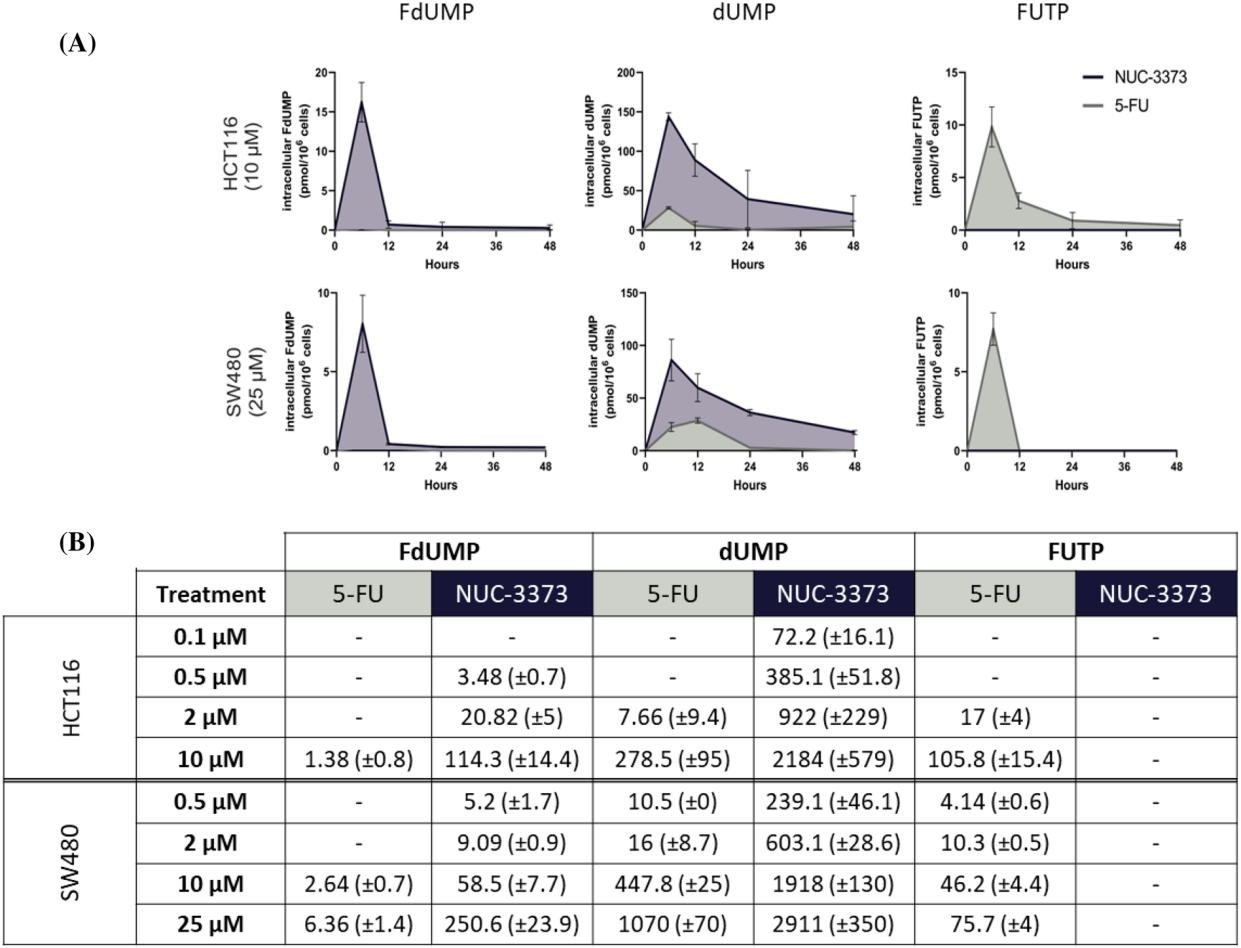
NUC-3373 and 5-FU are activated in CRC cells and inhibit TS. **A** Representative profile of intracellular metabolites related to 5-FU or NUC-3373 in HCT116 or SW480 cells treated with NUC-3373 or 5-FU for 6 h. Complete set of data available in supplementary information. **B** AUC values for FdUMP, dUMP and FUTP in HCT116 and SW480 cells treated with a range of concentrations of NUC-3373 or 5-FU. The data are expressed as (pmol x hour)/10^6^ cells ± SD (*n* = 3). Values were below the limit of quantification in cells marked with—(LLOQ FdUMP: 5 nM; dUMP: 50 nM; FUTP: 1 nM). dTMP and FdUTP could not be measured in any conditions tested as the levels were below limit of detection

As inhibition of TS by FdUMP results in increased dUMP and decreased dTMP [1, 18], intracellular nucleotide pools were assessed and quantified over time using mass spectrometry following 6-h treatment with NUC-3373 or 5-FU. NUC-3373 generated significantly higher levels of FdUMP compared to 5-FU. Free FdUMP was barely detectable following treatment with 5-FU in HCT116 cells, with an area under the curve (AUC) of 1.4 vs 114.3 at equimolar concentrations of 5-FU and NUC-3373, respectively and was only detected at a very low-level following treatment with 5-FU in SW480 cells, with AUC 6.36 vs 250.6 at 25 μM 5-FU and NUC-3373, respectively (Fig. 2). It is important to note that only free FdUMP was quantified, not FdUMP bound to TS.

NUC-3373 treatment resulted in a greater accumulation of dUMP metabolites suggesting greater inhibition of TS. The AUC was up to 120-times higher following treatment with NUC-3373 than 5-FU, at equimolar concentrations. However, while TS binding remained unchanged from 0.1 µM and up to 48 h, dUMP levels were dose-dependent and decreased over time after NUC-3373 was removed from the cell culture media. This suggests that new TS is being synthesized but not necessarily bound by the remaining intracellular FdUMP. Despite potential formation of new TS, and disappearance of FdUMP at 48 h even at high NUC-3373 doses, levels of dUMP remained at a minimum concentration of 20 pmol/10^6^ cells in SW480 cells treated with 25 µM NUC-3373, hence validating a prolonged inhibition of TS, unlike 5-FU. Furthermore, while 5-FU treatment led to generation of the metabolite FUTP in both cell lines, it was not detectable following treatment with NUC-3373 (LLOQ = 1 nM).

These results confirm that equimolar NUC-3373 leads to greater S inhibition than 5-FU and, consequently, increased levels of both dUMP and FdUMP, which will be converted to triphosphates that are likely to be misincorporated into DNA, but none or little of the fluorouridine metabolite FUTP which is misincorporated into RNA.

Thymidine supplementation rescued both cell lines from NUC-3373-induced cytotoxicity with IC_50_ > 100 µM, but it did not promote cell survival in 5-FU treated cells (Table 1), supporting the hypothesis that NUC-3373 is a more potent inhibitor of TS and DNA synthesis than 5-FU. Uridine triacetate is recommended for patients with severe side effects from 5-FU as it competes with FUTP for incorporation in RNA and limits unwanted toxicities [19, 20]. In our study, the addition of uridine resulted in an increased IC_50_ for 5-FU but had no effect on NUC-3373, indicating that misincorporation of FUTP into RNA is more important for the mode of action of 5-FU when administered over a short time period.

**Table 1:** Thymidine rescues cells treated with NUC-3373 from death, whereas uridine affects cell sensitivity to 5-FU

	5-FU—IC_50_ (µM)	NUC-3373—IC_50_ (µM)
HTC116 control	19.7 (15.1–24.9)	22.3 (19.6–25.6)
HTC116 + thymidine	15.8 (12.7–19.6)	> 100
HCT116 + uridine	27.3 (22.2–34.4)	22.9 (19.8–26.9)
SW480 control	44.6	67.9
SW480 + thymidine	34.8	> 100
SW480 + uridine	67.7	62.8

Data are represented as mean from at least three independent experiments, that each had six technical replicates (lower to upper 95% confidence interval, when applicable), maximal dose 100 µM

### NUC-3373 has a more DNA targeted mode of action than 5-FU

**Fig. 3:**
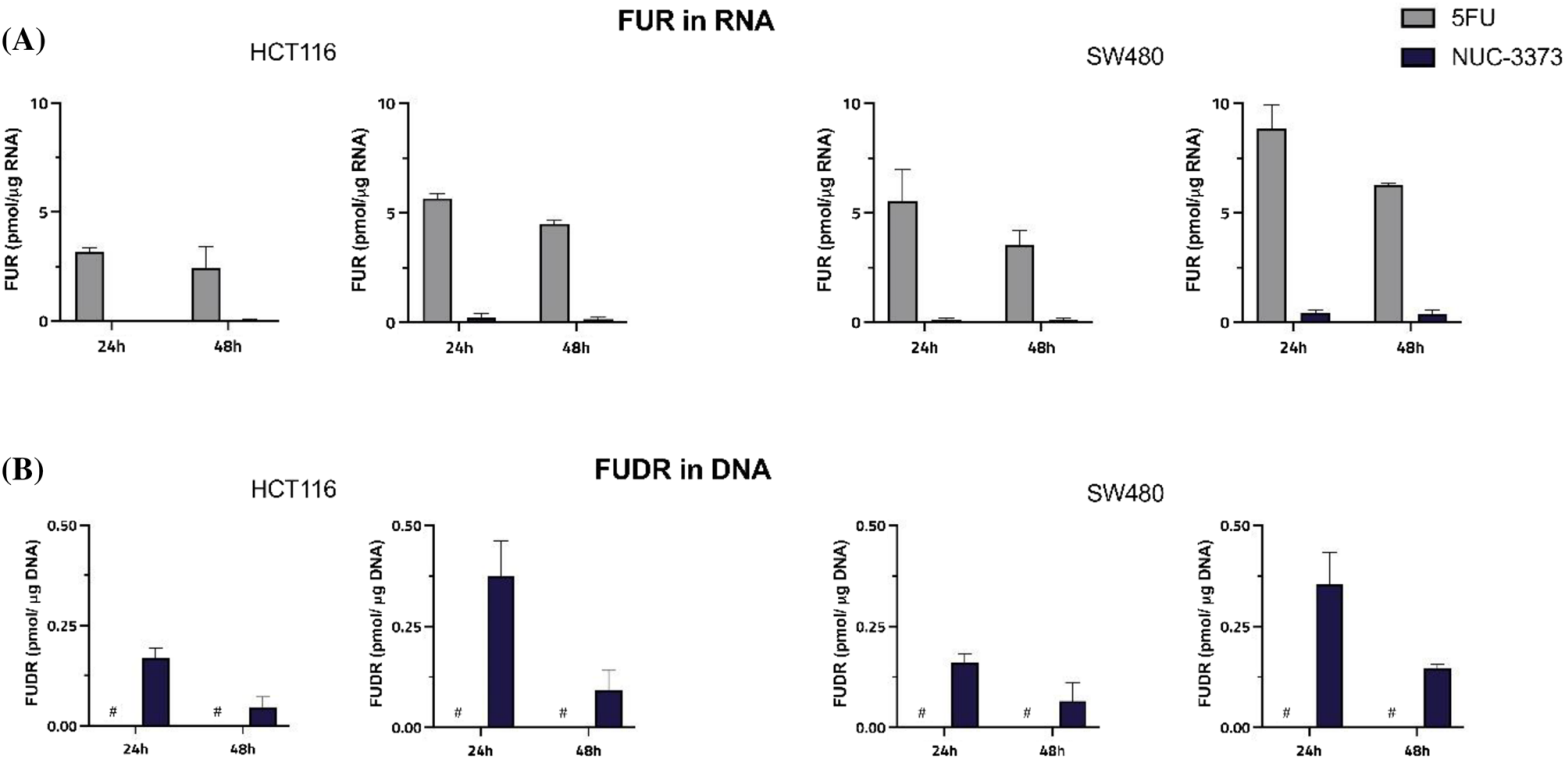
NUC-3373 mostly causes uracil misincorporation into DNA and treatment with 5-FU results in incorporation of uracil in RNA. HCT116 and SW480 cells were treated with 10–50 μM of 5-FU or NUC-3373. Incorporation of FUTP in RNA (**A**) and FdUTP in DNA (**B**) was determined with mass spectrometry, by measuring levels of FUR or FUDR. Data are represented as the amount (pmol) of FUR per μg RNA and FUDR per μg DNA ± SD (*n* = 3). # FUDR levels in 5-FU treated samples were below the lower limit of quantification (LLOQ = 0.1 nM)

FUTP is misincorporated in RNA during transcription and FdUTP into DNA during replication and repair processes [21]. Cells were treated for 24 h with 10–25 μM of NUC-3373 or 5-FU for HCT116 or 25–50 μM for SW480 and metabolites in the respective nucleic acids were quantified by mass spectrometry at 24 h and 48 h. Due to concerns regarding stability of monophosphate nucleotides for mass spectrometry analyses, a dephosphorylation step was included following the hydrolysis of RNA and DNA; therefore, the nucleosides fluorouridine (FUR) and fluorodeoxyuridine (FUDR) were used as surrogates for measurement of FUTP and FdUTP incorporation [22], respectively. Treatment with both 5-FU and NUC-3373 resulted in incorporation of FUTP in RNA (Fig. 3A). However, while FUR concentrations in 5-FU treated samples ranged from 2.44 to 8.84 pmol/µg RNA, they were at a maximum 0.5 pmol/µg RNA for NUC-3373. In contrast, cells treated with NUC-3373 demonstrated FUDR present in DNA, from 0.06 to 0.62 pmol/µg DNA, while no detectable signal was observed in DNA from cells exposed to equimolar doses of 5-FU (LLOQ = 0.1 nM) (Fig. 3B). These results support the hypothesis that a short infusion of 5-FU (≤ 24 h) causes FUTP generation, which is incorporated in RNA leading to the neutropenia, mucositis and gastrointestinal toxicities reported in patients. Conversely, NUC-3373 appears to have a more DNA-targeted mechanism of action, with minimal effects on RNA incorporation.

### dUTPase knockdown and biochemical assays confirm the DNA-mediated mode of action of NUC-3373

**Fig. 4:**
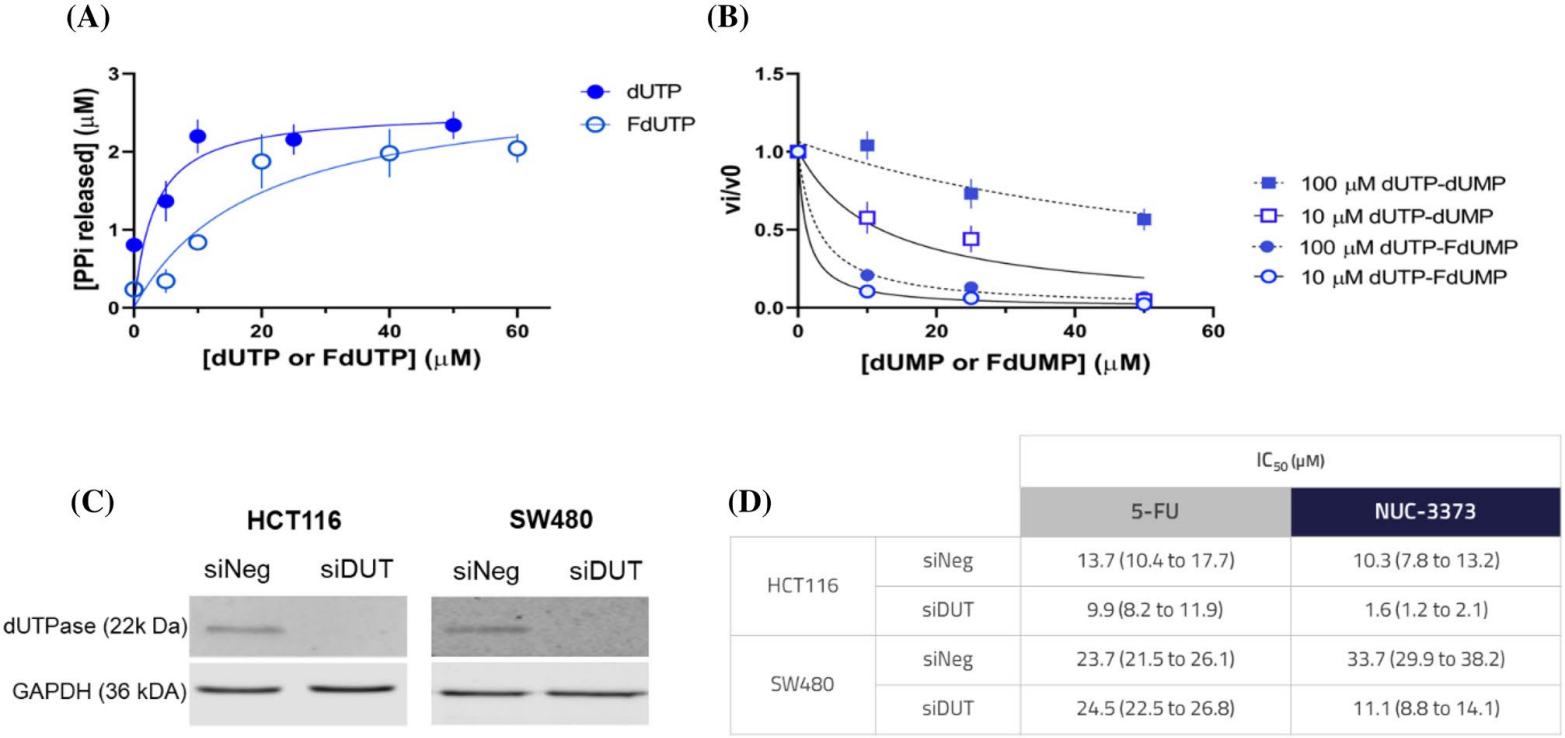
Lack of dUTPase validates action of NUC-3373 on DNA. **A** Activity assay for dUTPase using dUTP (filled circles) or FdUTP (empty circles). Data were fitted to a hyperbolic Michaelis Menten equation, yielding *K*_M-dUTP_ = 3.2 ± 1.8 μM, *K*_M-FdUTP_ = 19.1 ± 6.7 μM, *k*_cat-dUTP_ = 2.5 ± 0.3 μM, *k*_cat-FdUTP_ = 2.8 ± 0.4 μM. **B** Inhibition assay probing product inhibition with dUMP or FdUMP at *K*_M-dUTP_ and 10 times *K*_M-dUTP_ concentration of the substrate dUTP. Data were fitted using a Morrison’s equation and plotted as relative activity. Experiments were performed in triplicate and errors are shown as standard error of the mean. **C** dUTPase expression was silenced by siDUT and the efficacy of knockdown (72 h after transfection) was validated by western blot. GAPDH was used as loading control. **D** The effect of lack of dUTPase on sensitivity to NUC-3373 or 5-FU was assessed by measuring the IC_50_ (µM), compared to non-targeting siRNA control. Data are represented as mean from two independent experiments, each with six technical replicates (lower to upper 95% confidence interval)

Deoxyuridine triphosphate nucleotidohydrolase (dUTPase) prevents misincorporation of uracil into DNA by converting dUTP and FdUTP back into their monophosphate forms [23, 24]. Kinetic studies on dUTPase have suggested it is inhibited by its products dUMP or FdUMP. This would directly affect dUTP and FdUTP levels and therefore DNA incorporation [25]. To identify if product-mediated inhibition was taking place and perform a direct comparison between dUTP and FdUTP as substrates, we recombinantly produced human dUTPase and measured its activity using a coupled assay (Fig. 4A). dUTP was a better substrate than FdUTP (fivefold higher specificity, *k*_cat_/*K*_M-dUTP_ = 0.8 ± 0.4 μM ^−1^ s^−1^, *k*_cat_/*K*_M-FdUTP_ = 0.15 ± 0.06 μM^−1^ s^−1^). Furthermore, product inhibition occurred with both dUMP and FdUMP, being more extensive with accumulation of dUMP (inhibition constant 20-fold lower for FdUMP than dUMP, *K*_i-dUMP_ = 5.8 ± 1.4 μM and *K*_i-FdUMP_ = 0.62 ± 0.05 μM). Taken together, dUTP is a preferred substrate over FdUTP, and increased levels of both dUMP and FdUMP lead to product inhibition of dUTPase and consequently to greater incorporation of FdUTP into DNA.

High levels of dUTPase have been shown to negatively impact cell sensitivity to 5-FU, which was reversed upon silencing or blockade but only when cells were treated for 48 h [26] or 72 h [27]. This is consistent with the requirement for prolonged administration of 5-FU to cause uracil misincorporation into DNA. dUTPase expression was silenced in CRC cell lines by siRNA targeting the gene *DUT*, with a non-targeting siRNA control. The knockdown was confirmed by western blot at the time of treatment and validated up to 7 days post-transfection, when the endpoint cytotoxicity assay was performed (Fig. 4C, Fig. S4). SW480 cells expressed higher relative levels of dUTPase than HCT116 cells. This could explain the similarities in DNA misincorporation despite higher levels of dUMP and FdUMP in SW480 cells. Regardless of dUTPase expression, NUC-3373 exerted cytotoxic activity in both cell lines. Following knockdown, both cell lines were treated with a range of 5-FU or NUC-3373 concentrations and IC_50_ was determined 96 h post-treatment (Fig. 4D). A reduction of 67 to 83% in the IC_50_ of NUC-3373 was observed in cells transfected with DUT siRNA compared to cells transfected with siRNA negative control (in SW480 and HCT116, respectively), whereas knockdown did not affect sensitivity to 5-FU in either cell line. This confirms that incorporation of uracil in DNA is a key process underlying NUC-3373-induced cytotoxicity.

### TS inhibition and DNA incorporation cause cell cycle arrest and DNA damage

**Fig. 5:**
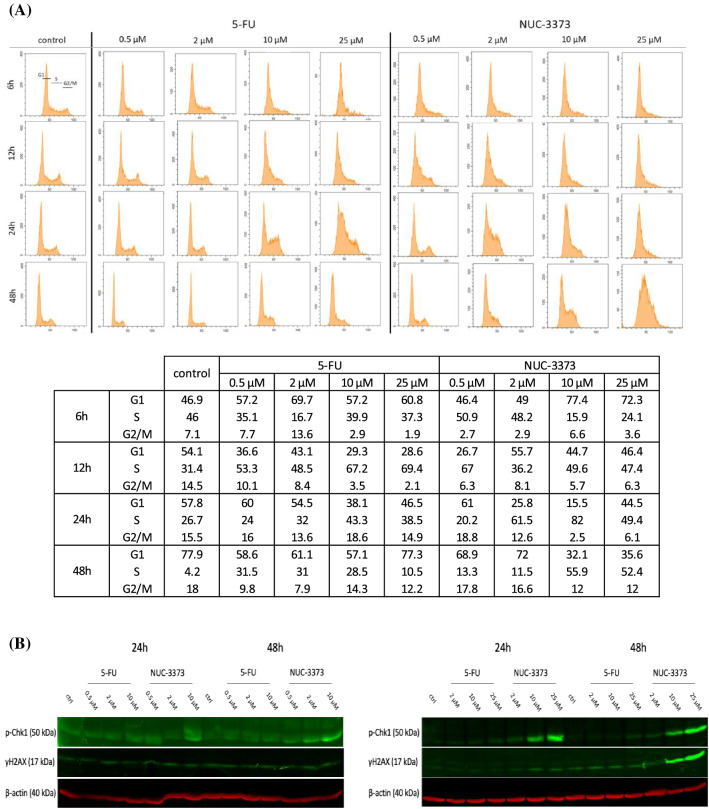
NUC-3373 induces a prolonged S-phase arrest and DNA damage. **A** SW480 cells were treated with sub-IC_50_ doses of 5-FU or NUC-3373 for 6 h and effect on the cell cycle was analyzed by flow cytometry at different timepoints. Gates for how each phase of a normal cell cycle (G1, S, G2/M) looks are represented in the corresponding control samples at 6 h and values are given in the table. Histograms and corresponding analysis from one representative experiment (complete dataset in Fig. S7). **B** Representative western blot images for γH2AX and p-Chk1 signals in HCT116 (left) and SW480 (right) cells treated with a range of 5-FU or NUC-3373 doses (0.5–25 μM), at 24 h and 48 h post-treatment. β-actin was used as a loading control (*n* = 2)

Nucleotide analogs disrupt the cell cycle when integrated into DNA, notably in S phase as they are usually misincorporated during replication [28], but less so during repair. We investigated the effect of 5-FU or NUC-3373 on the different phases of the cell cycle (Fig. 5A; Fig. S7). The proportion of cells in S-phase was greater following treatment with NUC-3373 compared to control and 5-FU at over a period of up to 48 h. At the highest doses, 5-FU treatment increased the proportion of cells in S-phase at 24 h, following which cells reverted to a regular cell cycle by 48 h. This suggests that NUC-3373 has a greater impact on DNA during replication than 5-FU and is consistent with the DNA incorporation data.

S-phase arrest can either be resolved by the cell to resume the cell cycle or it can induce strand breaks. Proteins involved in DNA repair such as histone H2A histone family member X (H2AX) and checkpoint kinase 1 (Chk-1) are phosphorylated (γ-H2AX and p-Chk1 respectively) and recruited to sites of DNA damage and are the initiators of downstream enzymes to restore DNA integrity [29]. To determine whether 5-FU and NUC-3373 cause DNA damage through the mechanisms described, expression of p-Chk1 and γH2AX were assessed over time in HCT116 and SW480 cells treated with sub-IC_50_ doses of 5-FU or NUC-3373 for 6 h. Both p-Chk1 and γH2AX were induced by NUC-3373 even at the lowest dose, resulting in a dose-dependent increase over time. The effect was more pronounced in SW480 cells, and while the western blot for HCT116 did not show a significant difference between the conditions, we had previously reported an increase of γH2AX signal 48 h post-treatment in cells treated with 0.5 or 10 μM NUC-3373 for 6 h [30]. Meanwhile, 5-FU had limited effect on either protein at equimolar doses in both cell lines (Fig. 5B). This further confirms that NUC-3373 causes DNA damage through TS and DNA synthesis inhibition, whereas 5-FU cytotoxicity is predominantly through an RNA-associated mechanism when administered over a short time period.

## Discussion

Thymidylate synthase is essential for conversion of dUMP to dTMP, representing the de novo pathway of dTMP generation for DNA replication and repair [31], making it an attractive target for anti-cancer therapy. Fluoropyrimidines have been developed to inhibit TS; however, these agents have drawbacks. NUC-3373 was specifically designed to overcome key limitations associated with 5-FU [7, 9]. The results of this study demonstrate that NUC-3373 generates substantially higher levels of the active anti-cancer metabolite FdUMP than 5-FU, suggesting it is a more potent inhibitor of TS. It is known that in vivo 5-FU is subject to extensive degradation by DPD in the liver, with only 15% of an administered 5-FU dose available to enter cancer cells [2]. Thus, the results are particularly encouraging as NUC-3373 and 5-FU were compared at equimolar concentrations in these in vitro experiments, meaning that cells were exposed to a higher percentage of 5-FU than they would receive in vivo. Furthermore, NUC-3373 did not generate high concentrations of the metabolite FUTP, which is known to be misincorporated into the RNA of both normal and cancer cells and underlies many of the gastrointestinal and hematological toxicities associated with 5-FU [32, 33]. These results are consistent with the observation that patients treated with NUC-3373 alone or in combination with oxaliplatin or irinotecan have experienced much lower rates of FUTP-related toxicity [34, 35]. The inhibition of TS combined with misincorporation of FdUTP in DNA and subsequent DNA damage prove that, unlike 5-FU, NUC-3373 is more effective at killing cells through targeting DNA rather than RNA.

In this study, two human CRC cell lines were studied based on their sensitivity to fluoropyrimidines. This allowed investigation of the potential impact of basal TS levels, cell replication rate, MMR and microsatellite status. TS levels vary widely in the tumors of patients with CRC [36] and, although the literature regarding TS as a predictive and prognostic biomarker for 5-FU is inconclusive, some studies show a correlation between TS expression and 5-FU activity [36–39]. However, we have previously shown cell sensitivity to NUC-3373 is independent of TS expression in a panel of CRC cell lines [38]. Patients receive 5-FU in combination with the folinic acid leucovorin to enhance the inhibition of TS by FdUMP, however, this did not reflect in our in vitro assays. Similar findings had already been reported by Dominijanni and Gmeiner [40] as they found that co-treatment with leucovorin had no significant effect on 5-FU cytotoxicity in HCT116 cells. Overall, it is not uncommon for research studies on 5-FU to not use leucovorin in vitro [16, 23, 26].

The difference observed in the dynamics of free and TS ternary complex formation at equimolar doses of 5-FU and NUC-3373 is due to the fact that 5-FU requires a complex activation pathway which results in an unpredictable metabolite profile [2, 18]. Therefore, either higher doses of 5-FU or prolonged treatment times are needed to achieve comparable results to NUC-3373 in these experiments. The kinetics of activation of both agents was investigated using a short 6-h treatment period to mimic a short infusion of 5-FU to compare with NUC-3373 which is administered as a short infusion to patients. This resulted in an unequivocal difference between 5-FU and NUC-3373, which was consistent in both cell lines. NUC-3373 generated more free FdUMP and no detectable FUTP, whereas the opposite was observed for 5-FU with barely detectable FdUMP. NUC-3373 also caused a greater increase in dUMP levels, which was used as a surrogate for TS inhibition, complementing the western blot data. In both cell lines, supplementation with exogenous thymidine rescued cytotoxicity caused by NUC-3373 but not by 5-FU, strongly indicating that NUC-3373 causes significant TS inhibition. When the de novo pathway of dTMP production is inhibited, cells can utilize the salvage pathway, converting thymidine to dTMP, to overcome thymine depletion [38]. On the contrary, supplementation with uridine, a method approved by the US Food and Drug Administration for patients who received an overdose or are exhibiting early-onset severe toxicities following administration of 5-FU [18, 37, 41], induced a shift in IC_50_ of 5-FU but not NUC-3373, implying further differences in the mode of action.

Upon entering S phase, TS translocates to the nucleus, directing dTMP synthesis to the site of DNA replication; however, TS ternary complex formation with FdUMP stops this translocation. It was previously reported that misincorporation of uracil into DNA was heightened when de novo dTMP synthesis was restricted to the cytoplasm [42], likely due to reduced dTMP pools available in the nucleus. We previously found that NUC-3373 induced cytoplasmic retention of TS [43]. While we could not measure dTMP levels, we observed increased FUDR misincorporation into DNA, leading to S-phase arrest in CRC cells and DNA damage, which was less pronounced in cells treated with 5-FU.

Uracil misincorporation into DNA and RNA has been investigated for decades, however, progress in analytical instruments now allows for more precise measurements. A recent study showed that HCT116 cells treated with 10 and 50 µM of 5-FU or FUDR for 24 h had similar levels of FUR incorporation into RNA with the two compounds, suggesting that FUDR is transformed back into 5-FU [22]. Furthermore, they could not detect any FUDR in DNA at less than 50 µM 5-FU and 30 µg DNA, whereas in our work it was present in as low as 4 µg DNA from cells treated with 10 µM of NUC-3373 for 24 h. This confirms that FdUMP released intracellularly by NUC-3373 accumulates and is further converted to FdUTP. The same group also found that ribosomal RNA (rRNA), the most abundant RNA species, is also affected by 5-FU. They observed that 5-FU treatment results in uracil incorporation in the ribosome, modifying its intrinsic translational activity, leading to increased translation of proteins involved in pathways controlling cell proliferation and tumorigenesis [44]. Incorporation of FUR in rRNA was also reported in samples from patients treated with 5-FU [45]. Therefore, fluorinated ribosomes induced by 5-FU treatment may favor the emergence of resistant cellular phenotypes and cause relapse. dUTPase minimizes misincorporation of uracil in DNA by maintaining the intracellular dUTP pool at an extremely low level [46, 47]. FdUTP is also a substrate for dUTPase and therefore these two nucleotides are often undetectable or present at very low concentrations in cancer cells under physiological conditions, or following treatment with 5-FU, as they are quickly converted to their respective monophosphates [23, 24]. Expression of dUTPase is heterogenous in tissues but increased expression is observed in various cancers, and it has been suggested that higher expression results in resistance to 5-FU [23, 24]. However, we demonstrated that dUTPase can be inhibited by its products dUMP and FdUMP, and that treatment with NUC-3373 resulted in an excess of both metabolites in CRC cell lines. This may explain why treatment with NUC-3373 resulted in FUDR being detected in DNA at similar levels at equimolar doses in both cell lines, even though SW480 cells have higher dUTPase expression compared to HCT116. Upon silencing dUTPase, NUC-3373-induced cytotoxicity was more pronounced in HCT116 than SW480, suggesting that dUTPase does not correlate with cell sensitivity and other mechanisms are involved in rescuing cells from death. Uracil DNA glycosylase (UDG) acts with dUTPase to maintain DNA free from uracil. UDG is part of the base excision repair (BER) pathway which removes a faulty base and aims to replace with the right one, but if no dTMP is available then it is likely to incorporate another uracil base instead and perpetuate the cytotoxic effect of fluoropyrimidine compounds [48, 49]. Yan et al*.* mapped cell lines based on their sensitivity to FUDR and p53 status and found two clusters: p53 wild-type were sensitive and p53 mutant were resistant to FUDR [49]. HCT116 cells are p53 wild-type and mismatch repair deficient, whereas SW480 cells have a mutated form of p53 and are mismatch repair proficient. These parameters may further explain differences in sensitivity, despite misincorporation of similar levels of FdUTP in their DNA. Wilson et al*.* showed that oxaliplatin-induced p53 inhibited dUTPase activity, using HCT116 p53 wild-type and HCT116 p53-/- cell lines as models [26]. NUC-3373 is currently being investigated in combination with oxaliplatin in the NuTide:302 study and this mechanism could partially explain the synergy previously observed between NUC-3373 and oxaliplatin in vitro. Up to 40% of patients with CRC are p53 wild-type [50, 51] and could benefit from this combination.

This study has demonstrated that in vitro, in addition to being a more potent TS inhibitor than 5-FU, NUC-3373 has a more DNA-targeted mode of action which may underly the advantages observed over 5-FU, such as a shorter infusion time and more tolerable safety profile. NUC-3373 may be an attractive alternative to one of the most commonly used chemotherapies for the treatment of colorectal and other solid tumors. These hypotheses will be further evaluated in a randomized phase 2 clinical study in which NUC-3373 will be compared against 5-FU in combination with agents commonly used in CRC.

## Supplementary Information

Below is the link to the electronic supplementary material.

Supplementary file1 (PDF 1176 KB)
