# Supplementary material for: The novel anti-cancer fluoropyrimidine NUC-3373 is a potent inhibitor of thymidylate synthase and an effective DNA-damaging agent
Source: Cancer Chemother Pharmacol. 2023 Mar 31;91(5):401–12. doi: 10.1007/s00280-023-04528-5 (PMC10156769; doi:10.1007/s00280-023-04528-5)
Supplement: Supplementary file 1 — Supplementary file1 (PDF 1176 KB) [file 280_2023_4528_MOESM1_ESM.pdf]

Supplementary information

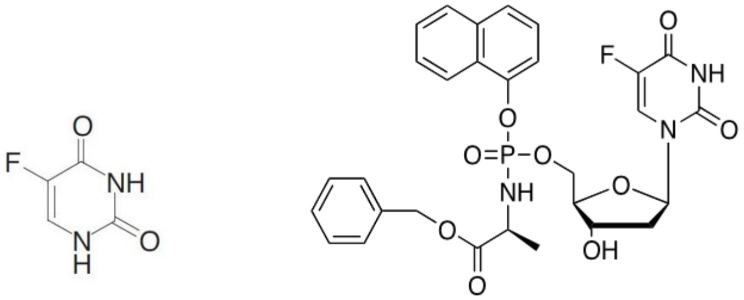

Figure S1. Chemical structure of 5-FU (left) and NUC-3373 (right). 5-FU has a fluor atom on the 5' position of uracil. NUC-3373 is composed of FdUMP and a phosphoramidate moiety (ester, aryl and amino acid)[1].

	HCT116	SW480
Sensitivity to fluoropyrimidines	Sensitive	Less sensitive
NUC-3373 IC_{50}	25 µM	45 µM
5-FU IC_{50}	15 µM	40 µM
Relative doubling time	Fast	Slow
Basal TS expression	Higher	Lower
MMR status	deficient	proficient
Microsatellite status	MSI	MSS

Table S1. CRC cell lines characterization. These two cell lines were chosen based on their difference in sensitivity to 5-FU and NUC-3373, their relative doubling time, their basal TS expression and MMR status.

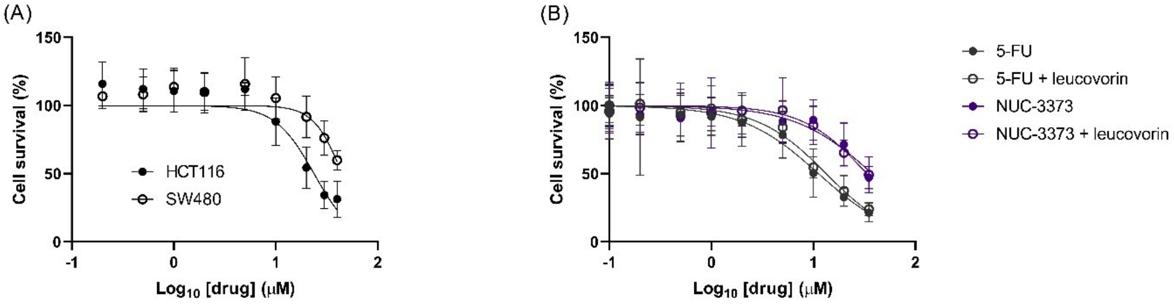

Figure S2. IC_{50} curves for NUC-3373 in colorectal cancer cell lines. (A) HCT116 and SW480 cells were co-treated with 5-FU or NUC-3373 for 24h and cell death was assessed at 96h post-treatment. (B) HCT116 cells were treated with 5-FU or NUC-3373 +/- 10 µM leucovorin for 24h and cell death was assessed at 96h post-treatment.

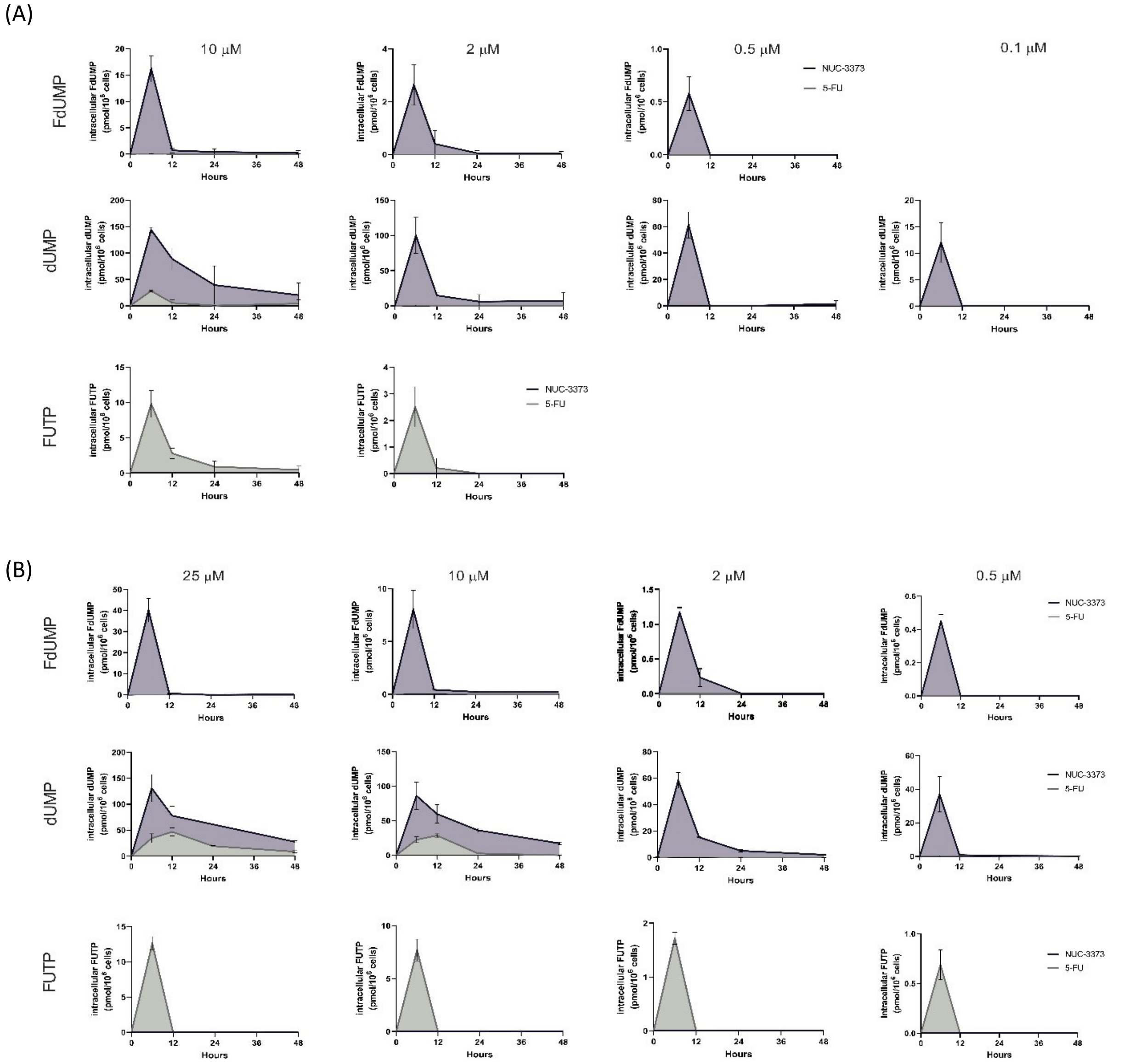

Figure S3. Metabolite profiles of CRC after treatment with 5-FU or NUC-3373. Full panel of metabolite profiles for (A) HCT116 and (B) SW480 cells treated with a range of concentrations of 5-FU or NUC-3373 for 6 hours. Samples were collected at 6-, 12-, 24- and 48-hours post-treatment. The data is expressed as pmol/10^6 cells +/- SD (n=3).

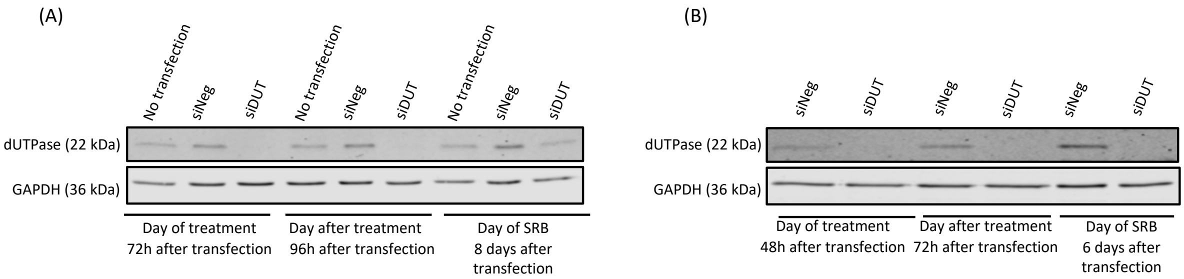

Figure S4. dUTPase knockdown validation at different timepoints in HCT116 and SW480. Forward transfection was performed for HCT116 (A) and reverse for SW480 (B) cells, knockdown was confirmed by western blot at different timepoints that correspond to time of treatment, time when the drugs were removed and time when the endpoint cytotoxic assay (SRB) was performed. No transfection cell lysate was used as extra control for HCT116 cells.

(A)

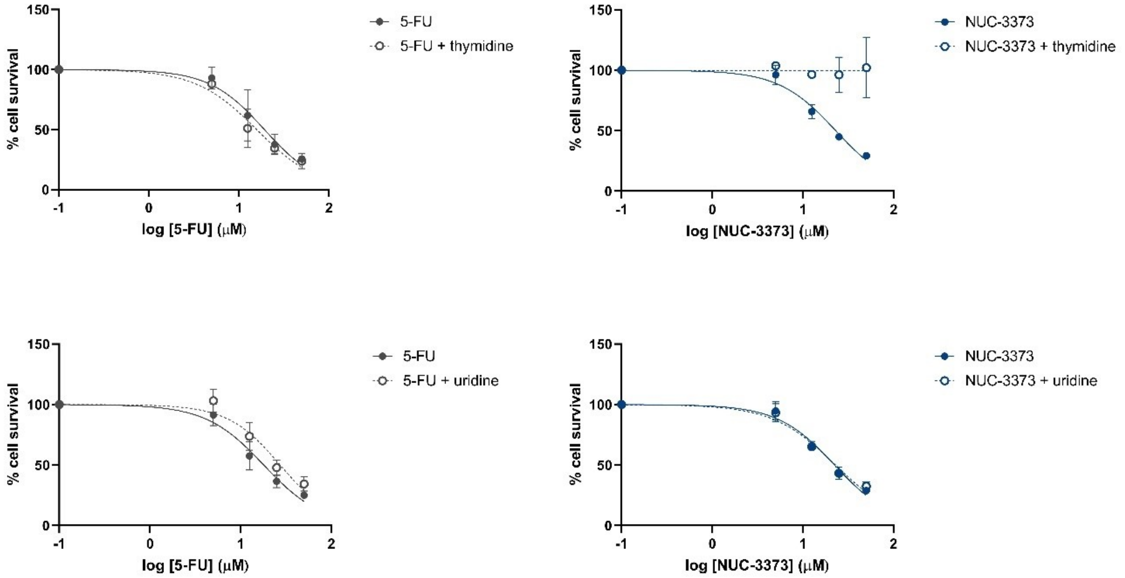

(B)

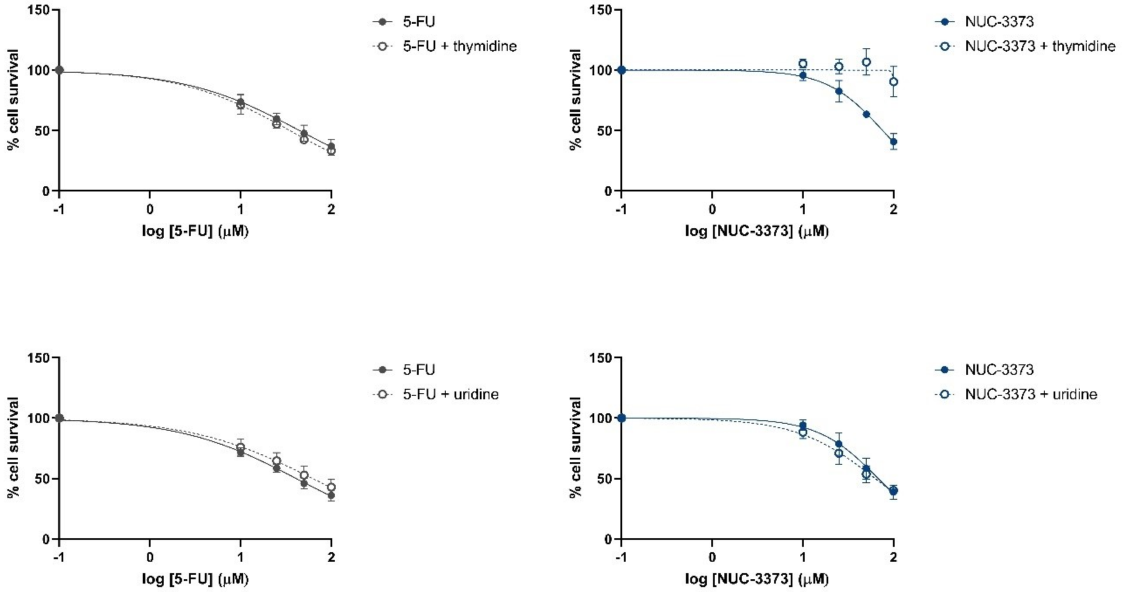

Figure S5. Dose-response curves for thymidine and uridine supplementation cytotoxicity assays. HCT116 (A) and SW480 (B) cells were co-treated with 5-FU or NUC-3373 +/- uridine or thymidine for 24h and cell death was assessed at 96h post-treatment.

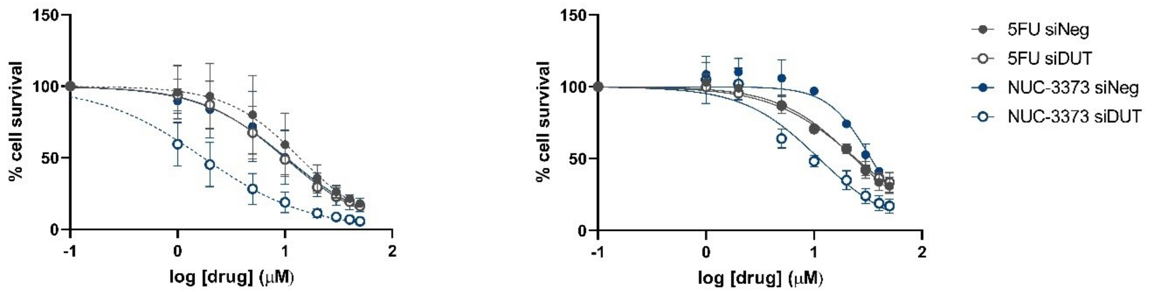

Figure S6. Dose-response curves for dUTPase knockdown cytotoxicity assays. HCT116 (left) and SW480 (right) were transfected with siRNA targeting *DUT* or non-targeting controls and then treated with 5-FU or NUC-3373 for 24h. Cell death was assessed at 96h post-treatment.

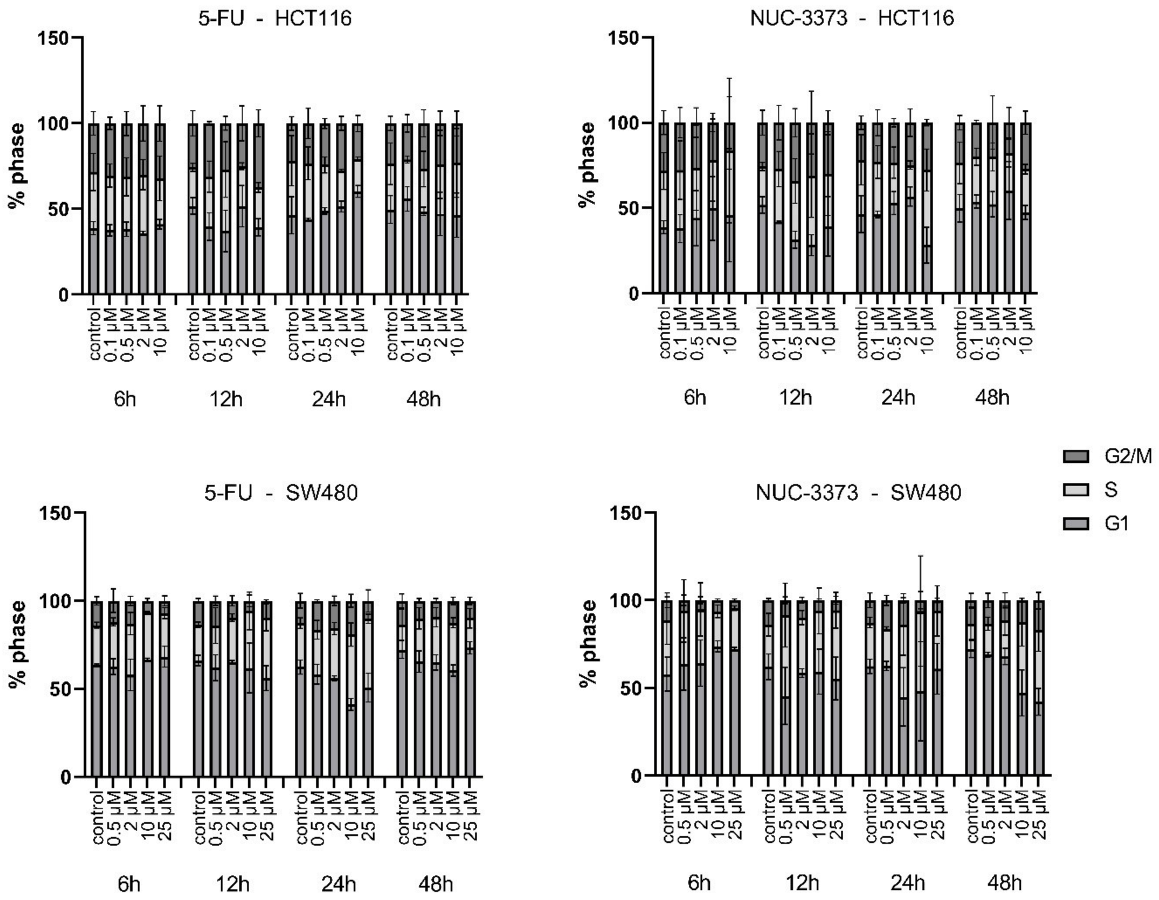

Figure S7. Cell cycle analysis. HCT116 and SW480 cells were treated with sub-IC_{50} doses of 5-FU or NUC-3373 for 6 hours and effect on the cell cycle was analyzed by flow cytometry at different timepoints. The graphs represent each phase of the cell cycle for the different conditions (n=3).

Supplementary material and methods

Sample preparation for metabolites incorporated into DNA and RNA

DNA and RNA were extracted from cells using Qiagen kits QIAamp DNA and RNeasy, according to manufacturer's protocols. The elution step for each was performed with 30 to 50 µL LC-MS grade water and nucleic acids were quantified with nanodrop. 150 µL of hydrolysis mix (250 U deoxyribonuclease (Roche), 300 mU phosphodiesterase I (Sigma-Aldrich) and 200 U alkaline phosphatase (Invitrogen) in 5 mL Tris-HCl buffer (20 mM, pH 7.9) containing 100 mM NaCl and 20 mM $MgCl_2$) was added to 4 to 10 µg DNA in 0.2 mL Eppendorf tubes. For RNA, 5 µg was incubated with 150 µL hydrolysis mix (5 U of calf intestine phosphatase (Promega) in 100 mM Tris-HCl, 50 mM NaCl and 10 mM $MgCl_2$). Samples were then incubated at 37°C overnight and kept in -20°C until analysis. Calibration standards were prepared for FUDR and FUR across the range 0.1- 20 nM and 0.05- 20 nM, respectively. Samples were mixed by vortex and evaporated to dryness under a stream of nitrogen gas. Samples were reconstituted in 100 µL of LC-MS-grade water and analyzed by LC-MS.

Chromatographic parameters

Triphosphate analysis (FUTP) was carried out on a Waters Atlantis Premier BEH C_{18} AX 1.7 µm, 2.1 mm X 100 mm column. A flow rate of 300 µL min^{-1} at initial conditions 90% 10 mM ammonium acetate pH 6 with 10% ACN until 0.5 min followed by a step change to 50% 10 mM ammonium acetate pH 6, 40% 10 mM ammonium acetate pH10 and 10% ACN. Re-equilibration to 90% 10 mM ammonium acetate pH 6, 10% ACN to 7 min. Monophosphate analysis (FdUMP and dUMP) was carried out on a Waters UPLC Premier HSS T3 1.8 µm, 2.1 mm X 100 mm column using a gradient method. Flow rate was set to 300 µL min^{-1} with initial conditions of 90% water + 0.1 % formic acid and 10% MeOH were held for 1.0 min followed by a linear gradient to 60% MeOH over 3 minutes and to 99% MeOH to 6 minutes. Pro-Tide analysis (NUC-3373) on was carried out on a Waters UPLC BEH C_{18} 130Å, 1.7 µm, 2.1 mm X 50 mm column. Flow rate was set to 300 µL min^{-1}, with 70% Water + 0.1% formic acid with 30% Acetonitrile + 0.1% formic acid held for 0.5 min followed by a linear gradient to 2.5 minutes to 99% Acetonitrile + 0.1% formic acid. Nucleoside analysis (FUDR and FUR) was carried out on an ACE Excel 2 AQ, 100 X 2.1 mm. Flow rate was set to 200 µL min^{-1} with 99% water + 0.1% formic acid with 1% Acetonitrile + 0.1% formic acid held for 1 min followed by a linear gradient over 2.5 minutes to 99% Acetonitrile + 0.1% formic acid and 1% water + 0.1% formic acid. Column temperature for each run was set to maintain 40°C.

Table S2. Mass spectrometric parameters

Analyte	Polarity	Mode	Ion	Ion(s) monitored (*m/z*)
FUTP	Negative	TOF	$[M-H]^-$	500.9518 (± 0.05 Da)
dUMP	Negative	MRM	$[M-H]^-$	307.0337→ 195.0028 (± 0.05 Da)
FdUMP	Negative	MRM	$[M-H]^-$	325.0243→ 129.0028 (± 0.05 Da)
NUC-3373	Positive	MRM	$[M+H]^+$	614.1698→ 262.0645 (± 0.05 Da)
FUR	Negative	MRM	$[M-H]^-$	261.0528→ 171.0190 (± 0.05 Da)
FUDR	Negative	TOF	$[M-H]^-$	245.0579 (± 0.05 Da)

Expression and Purification of dUTPase

A synthetic gene encoding the dUTPase isoform 2 (*Homo sapiens*, Uniprot code P33316) was ordered from Integrated DNA Technologies (IDT) as a GBlock and cloned into a pJ411 expression plasmid with a cleavable 6-histidine tag at N-terminus. The human dUTPase was transformed into *E.coli* BL21(DE3) cells for overexpression by culturing in LB medium with 50 µg/ml kanamycin at 37 °C 180 rpm shaking until the cells reached the OD_{600} = 0.8. Then, protein expression was induced with 0.5 mM of IPTG for overnight at 16 °C with shaking (180 rpm). Cells were harvested by centrifugation at 12000g, 4 °C, resuspended in buffer A (50 mM HEPES, 250 mM NaCl, 0.5 mM DTT and 30 mM imidazole, pH 7.5) with 1 µg/ml DNase and lysed using a cell homogenizer (Constant Systems). After centrifugation at 51000g for 30 minutes, 4 °C the supernatant was filtered with a 0.8 mm filter to remove particulates. This was loaded onto a 5 mL HisTrap column pre-equilibrated with buffer A followed by 10 column volumes wash before the elution. The dUTPase was eluted with buffer B (50 mM HEPES, 250 mM NaCl, 0.5 mM DTT and 500 mM imidazole, pH 7.5). Following elution, fractions containing dUTPase were pooled and dialysed with buffer C (50 mM HEPES, 250 mM NaCl, pH 7.5) and 2 mg/ml Tobacco Etch Virus (TEV) protease overnight. The dialysed mixture was loaded onto a 5 mL HisTrap column and the flow-through fractions were collected and analyzed by 15% SDS-PAGE. Fractions containing pure dUTPase (>95%) were pulled, flash frozen and used in the subsequent experiment. The yield of recombinant dUTPase was around 66 mg per litre of *E.coli* culture.

Sequence for protein expressed was as below, where the region in red is removed after cleavage with TEV protease:

MHHHHHHDYDIPTTENLYFQGMPCSEETPAISPSKRARPAEVGGMQLRFARLSEH
ATAPTRGSARAAGYDLYSAYDYTIPPMEKAVVKTDIQIALPSGCYGRVAPRSGLAAK
HFIDVGAGVIDEDYRGNVGVVLFNFGKEKFEVKKGDRIAQLICERIFYPEIEEVQALD
DTERGSGGFGSTGKN

siRNA-mediated knockdown of dUTPase

Cells were transfected with ON-TARGET plus Smart Pool siRNA against human DUT (siDUT, L-010258-00-0005, Horizon Discovery) or ON-TARGET plus Non-targeting Control Pool (siNeg, D-001810-10-05, Horizon Discovery) using Lipofectamine RNAiMAX (Thermo Scientific) following manufacture's instruction. For HCT116, forward transfection was performed. Cells were seeded in 6-well plate and reached around 60% confluent at time of transfection. siDUT/siNeg and Lipofectamine RNAiMAX were mixed in Opti-MEM (Gibco, Thermo Scientific) and incubated for 20 min at room temperature. The transfection mixture was then added to HCT116 cells to achieve a final siRNA concentration of 10 nM. After 24h, transfected cells were reseeded at 1000 cells/well into 96-well plates for following process. For SW480, reverse transfection was performed. siDUT/siNeg and Lipofectamine® RNAiMAX were mixed in Opti-MEM and incubated for 20 min at room temperature. In parallel, SW480 cells were trypsinized and resuspended to a cell density of 3.6×10^4 cells per mL of medium, and RNAiMAX/siRNA/Opti-MEM (200 µL per mL) was added to achieve a final siRNA concentration of 8.33 nM. The cell suspension was mixed gently and the cells were seeded at 3000 cells/well into 96-well plates for following process. Knockdown of dUTPase was confirmed by western blot, using dUTPase antibody (Abcam #229122, 1:1000).

1. Mcguigan C, Murziani P, Slusarczyk M, et al. Phosphoramidate ProTides of the Anticancer Agent FUDR Successfully Deliver the Preformed Bioactive Monophosphate in Cells and Confer Advantage over the Parent Nucleoside. *J Med Chem*. 2011;54:7247-7258. doi:10.1021/jm200815w
